# Increasing Energy Flux to Maintain Diet-Induced Weight Loss

**DOI:** 10.3390/nu11102533

**Published:** 2019-10-21

**Authors:** Christopher L. Melby, Hunter L. Paris, R. Drew Sayer, Christopher Bell, James O. Hill

**Affiliations:** 1 Department of Food Science and Human Nutrition, Colorado State University, Fort Collins, CO 80523, USA; 2 Division of Natural Sciences, Pepperdine University, Malibu, CA 90263, USA; 3 Department of Nutrition Sciences, University of Alabama-Birmingham, Birmingham, AL 35294, USA; 4 Department of Health and Exercise Science, Colorado State University, Fort Collins, CO 80523, USA

**Keywords:** energy intake, energy expenditure, body weight regulation, appetite, exercise, weight loss

## Abstract

Long-term maintenance of weight loss requires sustained energy balance at the reduced body weight. This could be attained by coupling low total daily energy intake (TDEI) with low total daily energy expenditure (TDEE; low energy flux), or by pairing high TDEI with high TDEE (high energy flux). Within an environment characterized by high energy dense food and a lack of need for movement, it may be particularly difficult for weight-reduced individuals to maintain energy balance in a low flux state. Most of these individuals will increase body mass due to an inability to sustain the necessary level of food restriction. This increase in TDEI may lead to the re-establishment of high energy flux at or near the original body weight. We propose that following weight loss, increasing physical activity can effectively re-establish a state of high energy flux without significant weight regain. Although the effect of extremely high levels of physical activity on TDEE may be constrained by compensatory reductions in non-activity energy expenditure, moderate increases following weight loss may elevate energy flux and encourage physiological adaptations favorable to weight loss maintenance, including better appetite regulation. It may be time to recognize that few individuals are able to re-establish energy balance at a lower body weight without permanent increases in physical activity. Accordingly, there is an urgent need for more research to better understand the role of energy flux in long-term weight maintenance.

## 1. Introduction

Decades of research have shown that calorie-restricted diets of any macronutrient composition rarely result in permanent weight loss, and that long-term success rates for obesity treatment (apart from bariatric surgery) are low [1]. In light of this phenomenon, the U.S. National Institutes of Health sponsored a 2019 workshop titled, The Physiology of the Weight-Reduced State, for the purpose of addressing the issue of poor long-term obesity treatment outcomes. Discussions at this workshop centered on the contributions of genetics and the environment to the epidemic of obesity, as well as the physiological adaptations, behaviors/habits and the obesogenic environment interacting to present significant barriers to long-term weight loss. There was general consensus that energy restriction leading to the weight-reduced state is characterized by decreased total daily energy expenditure and increased hunger. Moderators called for greater research efforts focused on creative approaches to overcome these barriers such that energy balance and maintenance of the lost body mass (primarily fat mass) can be achieved in the face of these metabolic adaptations that are at odds with the current obesogenic environment. 

In this paper, we discuss different ways that energy balance can be achieved and the potential consequences for longer term body weight regulation with special attention to the weight-reduced state. We focus on the concept of energy flux from a theoretical perspective, the components of energy flux, the impact of various modes and intensities of exercise on energy flux, and the potential advantages of achieving post-weight loss energy balance and weight maintenance at higher levels of energy intake and expenditure (high energy flux). This is not meant to be an exhaustive review, but rather to propose that based on evolutionary biology and high food availability in our current environment, high energy flux is inevitable, but how a high energy flux state is re-established following weight loss is critical to long term healthy weight management. 

## 2. How is Energy Flux Defined and Measured? 

The term ‘flux’ in biology refers to a relatively simple concept—the rate of movement of a substance, such as calcium ions, from one cellular compartment or tissue to another, which takes into account the magnitude and direction of flow (both efflux and influx) of the substance. Germaine to cellular bioenergetics, one can speak of ATP flux as ATP turnover, which involves the dual processes of ATP hydrolysis and synthesis required for biological work. In regard to body weight regulation, a number of different definitions and explanations for the concept of energy flux can be found in the scientific literature and these typically take a macroscopic, whole-body view. Unfortunately, the use of this term is inconsistent and has led to some confusion. Bell et al. [2] specified energy flux as the “absolute level of energy intake and expenditure under conditions of energy balance”. In a similar fashion, Goran et al. [3], Bullough et al. [4], and Hagele et al. [5] have used the term in experimental studies to describe ‘energy turnover’, occurring as a function of total daily energy intake (TDEI) matched to daily expenditure. Similarly, Swinburn et al. [6] have used the term to describe total daily energy expenditure (TDEE) based on doubly-labeled water studies with the assumption that during the several weeks of TDEE measurement, individuals were in energy balance and metabolizable energy from food was approximately equal to TDEE. Hume et al. [7] defined energy flux as habitual energy intake plus habitual energy expenditure and then later in the same paper they described energy flux as the ‘absolute level of energy balance’. In a more mechanistic description, Hand et al. [8] defined energy flux as “the rate of caloric conversion from initial absorption into the body tissues to utilization in metabolism or its transformation into energy stores”. Their definition, encompassing the process of transforming ingested energy into body energy stores, does not assume energy flux to be characterized by a state of energy balance. It is apparent then, that the use of the term ‘energy flux’ is not uniformly consistent and its relationship with energy balance is not consistently described. 

The magnitude of energy turnover in the body during a given time period is dependent on energy expenditure, independent of whether the energy provided is from ingested energy or endogenous stores. While it may be reasonable to define energy flux as TDEE in a steady state where metabolizable energy from food and energy expenditure are matched, it is also important to understand differences in how that energy expenditure is achieved. In other words, simply considering energy flux as total energy expenditure fails to consider the individual contributions of resting energy expenditure (REE), the thermic effect of feeding (TEF), exercise energy expenditure (ExEE), and non-exercise activity thermogenesis (NEAT). The latter two are the components of physical activity energy expenditure (PAEE). 

While low and high flux conditions have been previously described as energy balanced states with no net loss or gain of energy stores over time [2,3,4,6,9,10], one might argue that high and low flux states can occur at least initially, with some degree of energy imbalance. For example, an individual who expends a total of 10,460 kJ/day (2500 kcal/day), but only consumes 8368 kJ/day (2000 kcal) of metabolizable food energy, still has an energy flux of 10,460 kJ/day (at least initially). The energy requirement is readily met by the influx of 8368 kJ/day of metabolizable energy ingested and an additional 2092 kJ/day (500 kcal/day) drawn from the body’s energy stores. Thus, energy flux could be measured at any point in time as energy expenditure regardless of whether the energy comes from exogenous or endogenous sources. However, energy flux as a concept related to the maintenance of lost weight is likely to be more useful in reference to periods of weeks to months. Given that a goal of obesity treatment is maintenance of lost weight in a state of energy balance, when examining the role of energy flux in preventing weight regain, our working definition of energy flux is the magnitude of total energy turnover while maintaining energy balance over periods of weeks to months. Consistency in how the term is used among studies would be helpful in determining the level of importance this concept has for body weight regulation. 

Although energy flux is a function of intake (influx) and expenditure (efflux), these should not be summed to quantify flux, as this inflates the actual throughput of energy in the body [11]. The magnitude of energy flux can be expressed as both absolute and relative values. The former is synonymous with TDEE; the latter is expressed relative to body size or to REE, thus allowing comparisons between individuals with differing body sizes and REE values. One possibility is to express flux as a multiple of REE (TDEE/REE) [10], which has also been termed metabolic scope [12]. This approach provides a measure of flux that is synonymous with that of the commonly used physical activity level (PAL) used to describe the range of daily energy expenditures—from those of sedentary individuals to athletes. It is also similar to the concept of metabolic equivalents (METs, multiples of REE) used to quantify physical activity and exercise intensities as fold changes in energy expenditure relative to rest. Thus, an individual with a TDEE of 10,460 kJ/day (2500 kcal/d) and an REE of 6276 kJ/day (1500 kcal/d), would have an absolute energy flux of 10,460 kJ/day and a metabolic scope of 1.67 that represents relative energy flux. Using this approach, it may be possible to identify an optimum energy flux for an individual or a population. A similar approach might be to quantify energy flux as non-REE by subtracting REE from TDEE. However, REE is also the major contributor to TDEE (except for athletes who on some days are training extensively and exhibit extremely high levels of PAEE). So, a person with a high REE (as in the case of obesity) could have the same absolute energy flux as a smaller, highly physically active person with a much lower REE, but the contributors to high energy flux would be quite different and these metabolic states are also quite different. 

While acknowledging the inherent difficulties in arriving at a standard approach to measuring energy flux, we propose that energy flux should be quantified in both absolute (TDEE while in energy balance) and relative terms as metabolic scope (TDEE/REE while in energy balance). Quantifying absolute energy flux as TDEE demonstrates that similar levels of high flux are achievable by sedentary persons with large body mass and by physically active persons with much lower body mass. Quantifying relative energy flux demonstrates the different contributions of non-REE (primarily PAEE) to the magnitude of energy flux in these two individuals. This use of the metabolic scope as a relative measure of flux is an especially useful construct in regard to the weight-reduced state, as it focuses on the importance of increasing TDEE not by weight regain and associated increases in REE, but by increasing the contribution of PAEE to TDEE independent of increases in body mass. Furthermore, for reasons discussed later in our concept paper, we propose that in the weight-reduced state, achieving a metabolic scope of 1.7–1.8 would be a starting point for a “desired value”, although much more investigative work is required in this important research area.

## 3. Does Human Evolutionary Biology Interacting with the Current Obesogenic Environment Necessitate a High Energy Flux? 

Speakman [13,14] has proposed that based on evolutionary biology, selective pressures were exerted such that gene variants (alleles) were expressed in order to protect humans from starvation, disease and pathogen-induced anorexia, and predation. Thus, body size and composition were favored that would enhance survivability when food was scarce, when infectious disease risk was high, and also when needing to move quickly to escape predation. In other words, some body energy stores would be critical to survive periodic food deprivation and disease states, but excessive body stores would predispose one to greater predation risk. However, given that humans rarely face risk of predation today, genetic drift has occurred over time such that we are now protected more against weight loss than against weight gain. 

**Figure 1:**
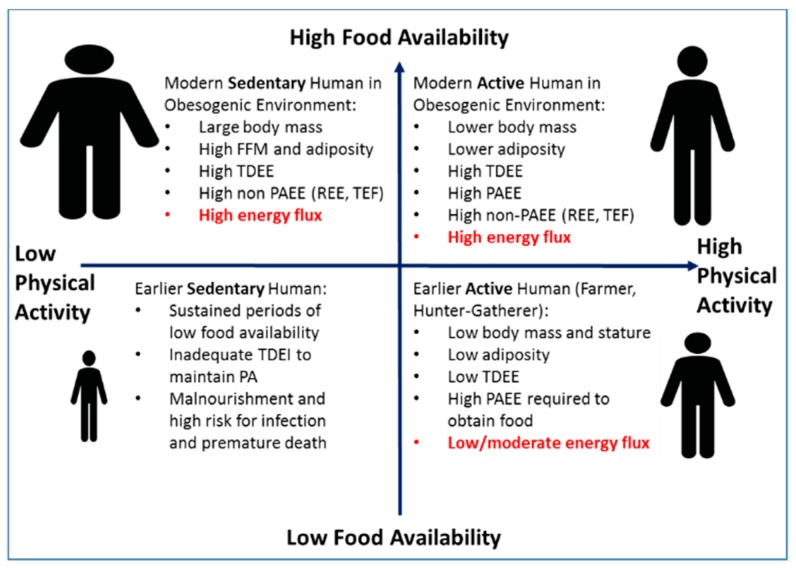
Total daily energy expenditure (TDEE) and body size are environmentally determined largely by the interaction of food availability (y-axis) and the need for physical activity (x-axis). Adapted from Pontzer [23].

There has been considerable interest in the possible roles of genetics in body weight regulation. Studies of monozygotic twins provide heritability estimates of up to 70–80% for various measures of body size [15,16]. In a well-controlled overfeeding study of monozygotic male twins, there was significantly greater concordance in weight gain and body composition changes within twin pairs compared to between twin pairs [17]. However, genome-wide association studies have provided little support for the role of genetics in determination of body mass index (BMI) at the population level [18,19]. Indeed, these population studies demonstrate only a small fraction of the variability in adult BMI values is explained by identified risk alleles. In a recent paper, Muller et al. [20] argue that the genetic basis for common obesity is not well established, and that body weight is not tightly regulated. They propose an alternative view that the recent increases in body weight and adiposity within the population primarily reflect physiologic adaptations to an affluent environment that favor energy balance being achieved at a higher body mass and level of adiposity. This argument is bolstered by ecological studies in humans [21] and non-human primates [22] demonstrating that TDEE and body size are environmentally determined largely by the interaction of food availability and the need to engage in physical activity (PA). These studies by Pontzer et al. [21,22,23,24], based on observation and evolutionary theory, strongly suggest that the availability of food is a principal driver of TDEE and therefore absolute energy flux if overall energy balance is achieved (e.g., high-food availability results in high flux). Indeed, in a large multicenter study, in which energy flux was measured using doubly-labeled water in almost 1400 adults, Swinburn et al. [6] observed a strong positive relation between TDEE and body weight, with TDEI as the driver of both TDEE and body weight. However, the mechanism by which a population achieves a given level of energy flux is determined by the interaction of the need for PA within the context of that specific food environment (Figure 1). Throughout most of human history, food availability was generally low or sporadic and the need to engage in PA for survival was high. These conditions resulted in populations, such as hunter-gatherers, with a lower absolute energy flux achieved through a combination of a small body size (to reduce energy costs of metabolically active tissues to enhance survival chances) and by reducing non-PA energy expenditure. In environments characterized as high-PA and higher food availability (e.g., subsistence farmers), a moderate level of energy flux was achieved by a high level of PA and maintaining a small body size [21]. Conversely, individuals living in low-PA/high-food availability environments (e.g., modern Western societies and captive animals) achieve high absolute energy flux primarily through increases in body size [21,23]. However, we propose that higher energy flux could also be achieved by greater PA at a smaller body mass. The combination of low food availability and low energy expenditure historically would occur with extreme conditions that are often not compatible with health. These findings have important implications related to the prevalence of obesity in the modern, low-PA/high-food availability environment and the relative importance of diet, PA, and energy flux for causing, preventing, and treating obesity within that inherent context. 

### Proposed Relationships among Physical Activity, Energy Flux, and Body Size

Over the past years, levels of PA have been declining [25] and public health strategies have been aimed at helping people reduce energy intake. While it is theoretically possibly to maintain energy balance at low body mass by matching a low TDEI with a low TDEE, the high rates of obesity in the U.S. and other populations and the fact that long-term weight loss is rare, suggest that this strategy is not working. These poor results are not unexpected given the virtually unlimited availability of easy-to-obtain, highly palatable, and energy dense food in many parts of the world. 

**Figure 2:**
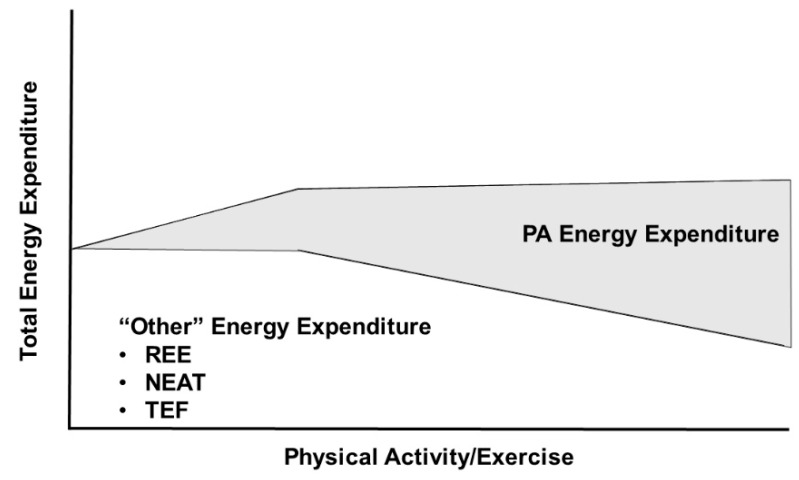
The Constrained Total Energy Expenditure Model suggests that total daily energy expenditure is maintained within a narrow range at high levels of physical activity energy expenditure by reducing other components of energy expenditure. Adapted from Pontzer et al. [21].

Central to the interacting relationships of food availability, environmental PA requirements, body size, and TDEE is the Constrained Total Energy Expenditure Model of Pontzer et al. [21,23] (Figure 2). According to this model, increasing physical activity increases TDEE only at lower levels of physical activity, but at higher levels of physical activity, total daily energy expenditure remains constant despite further increases in physical activity. In a demonstration of the constrained nature of the relationship between TDEE and PA, objective measures of both were determined by doubly labeled water and accelerometry, respectively, in 322 adults from five populations (Ghana, South Africa, Seychelles, Jamaica, and United States) [21]. These data demonstrated a breakpoint at 230 accelerometry counts per minute per day (CPM/d) where increased PA below this level resulted in a proportional increase in TDEE, but PA levels above this level did not result in further increases in TDEE. The 230 CPM/d breakpoint occurred at approximately the 70th percentile of PA in this cohort, i.e., the majority of adults had physical activity levels below the breakpoint. Pontzer et al. [21] hypothesize that increases in PA energy expenditure above this breakpoint are offset by reductions in non-exercise energy activity thermogenesis and, more importantly, non-muscular physiological functions such as processes related to reproductive activity and somatic maintenance. Pontzer et al. [23,24] have interpreted these data to suggest that increased exercise will have little utility in producing weight loss given the limited impact of increased PAEE on TDEE. Thus, an increase in PA will be unable to offset the effects of high TDEI associated with high food availability. Instead, the high TDEI will result in weight gain with the attendant increase in energy flux resulting from elevated non-PAEE. It follows that efforts to reduce energy intake would be ineffective in individuals within a high-food availability environment that strongly favors (or even necessitates) a state of high energy flux. 

## 4. Energy Flux: Does It Matter How It Is Attained?

**Figure 3:**
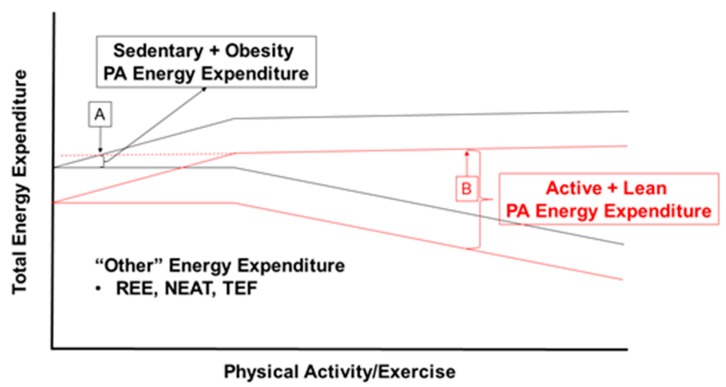
Theoretical scenarios where total daily energy expenditure and energy flux are constant between points A and B, but the contribution of physical activity energy expenditure is substantially different. Black and red lines represent regression lines for the Constrained Total Daily Energy Expenditure Model with no adjustment for body size. At point A, a high energy flux state is achieved through a sedentary lifestyle and large body size. At point B, high flux is achieved at a lower body weight and highly active lifestyle. Abbreviations: PA, physical activity; REE, resting energy expenditure; NEAT, non-exercise activity thermogenesis; TEF, thermic effect of feeding.

If the current environment, coupled with human evolutionary biology is driving individuals toward high TDEI, yet TDEE is constrained despite increases in physical activity, is there any alternative to achieving a high flux state by way of increased body mass? Contrary to conclusions by Pontzer, we suggest that the level of volitional PA could be a major determinant of body size within this high-food availability and high energy flux environment, especially in the weight-reduced state. In such an environment, high energy flux could be achieved by an active lifestyle and smaller body mass rather than through a sedentary lifestyle and large body size (e.g., obesity). Figure 3 demonstrates these theoretical scenarios within the context of the Constrained Total Energy Expenditure Model, whereby individuals can reach a similar level of energy flux through increased physical activity or large body mass. For example, a lean individual with an REE of 6700 kJ/day (1600 kcal/day) and a reasonably high level of PAEE could exhibit a high flux state at 11,715 kJ/day (2800 kcal/day) with a metabolic scope of 1.75 (11,715/6700 kJ = 1.75), whereas a sedentary individual with obesity and an REE of 8370 kJ/day (2000 kcal/day) could have the same absolute level of energy flux (TDEE = 11,715 kJ/day), but with a metabolic scope of 1.4 (TDEE/REE = 1.4). The way that the absolute energy flux is achieved between these two individuals is dramatically different. Whereas, a high flux state in obesity is driven by excessive TDEI leading to a large body mass, the principle driver of the high flux state in an active individual would be high levels of physical activity. In this sense, as alluded to earlier, it may be useful to define energy flux as more than just the level of energy expenditure with greater consideration to the principle drivers of the high flux states.

### 4.1. Metabolic Advantages of Achieving High Flux through High Levels of Physical Activity

Achieving high energy flux through physical activity is clearly associated with better metabolic function. This has been described by some as being metabolically flexible—the ability to match fuel availability and utilization throughout the day in response to changes in feeding and activity states [26]. Throughout much of human history, moderate amounts of total and visceral body fat were beneficial to survival. The readily mobilized non-esterified fatty acids (NEFAs) from visceral adipocyte triacylglycerols, in addition to providing fuel for immune cells, circulate directly to the liver and would have provided fuel to support hepatic gluconeogenesis from glycerol and glucogenic amino acids, thus affording maintenance of adequate circulating glucose during times of inadequate food availability. The flux of fatty acids into the liver from visceral fat depots and their subsequent oxidation to acetyl CoA would have also increased ketogenesis, another key to surviving periods of inadequate energy availability. These metabolic responses would have been important to enable significant physical activity required for obtaining food. Then in response to the return of food availability, the body’s metabolism would respond quickly to handle the overload of macronutrients, both by increased oxidation and storage. Thus, it seems likely that early humans maintained the metabolic flexibility and appetite regulation necessary to rapidly adjust and survive significant perturbations of fasting, feeding, and high levels of physical activity. 

#### Appetite Regulation

Mayer et al. [27] first suggested that high levels of PA are associated with more precise regulation of energy balance. His seminal work conducted in West Bengali mill workers demonstrated a U-shaped relationship between energy intake and PA requirements of the workers’ positions. Individuals in the most sedentary occupational groups consumed as much energy as those performing jobs requiring heavy and very heavy work, which resulted in higher body weights among sedentary workers compared to the most active workers. Here again, both groups of workers were in equivalent states of overall energy flux, but the relative contributions of body size and PA varied greatly. They concluded that energy intake is matched to energy expenditure only in the ‘normal activity range’, but below this range of activity, energy intake and expenditure are initially uncoupled, resulting in positive energy balance. 

Intuitively, higher daily energy expenditure characteristic of a high flux state would appear to result in better regulation of energy intake in the midst of an obesogenic environment, owing to the necessity of greater energy intake required to maintain energy balance. While some individuals may exercise so they can eat more food with less volitional restraint, the benefits of higher ExEE to body weight regulation extend beyond the opportunity for greater food indulgence. Indeed, Blundell and colleagues [28,29,30] have shown that increased physical activity is associated with greater meal-induced satiety and better appetite regulation. In a recent experimental study, Hagele et al. [5] reported that high energy flux over a 3–d period (metabolic scope of 1.8) from treadmill walking resulted in acutely greater appetite control, whereas low energy flux (metabolic scope = 1.3) resulted a positive energy balance of 17.5% during ad libitum intake.

### 4.2. Metabolic Disadvantages of Achieving High Flux through Increased Body Mass

The metabolic consequences of achieving high energy flux through increased body weight are essentially the opposite of those seen with achieving high energy flux through physical activity. Increasing body mass (developing obesity) is associated with metabolic inflexibility and sedentariness is linked with increased energy intake, which together may render individuals with overweight/obesity more susceptible to weight gain during acute periods of overfeeding [31]. 

In our current obesogenic environment, in which food deprivation is rare and physical activity is low, excessive visceral adiposity is common and detracts from metabolic flexibility. Many overweight/obese individuals are characterized by metabolic inflexibility, in which adjustments to changes in energy and macronutrient intake are met with sluggish metabolic responses that are associated with macronutrient overload, glucotoxicity and lipotoxicity characterized by hepatic and skeletal muscle lipid accumulation, dysfunctional adipose tissue, insulin resistance and metabolic disease. 

#### Appetite Dysregulation

Several recent studies provide further evidence of appetite dysregulation at low levels of PAEE. In a room calorimeter experimental study, Stubbs et al. [32] found that men expending 1.4 × REE (9700 kJ/day; 2320 kcal/d) or in a sedentary condition for seven consecutive days ingested as much metabolizable food energy as when they were more active, expending 1.8 × REE (12,800 kJ/day; 3060 kcal/day) for the 7 days in the calorimeter. They concluded that reducing physical activity fails to result in a compensatory decrease in energy intake. In a prospective longitudinal study of more than 400 individuals, Shook et al. [33] found that at baseline, the lowest compared to highest level of physical activity was associated with greater appetite dysregulation and significantly greater increases in fat mass over the one-year follow-up. 

## 5. How Does Physical Activity Influence Energy Flux? 

The quickest viable approach to changing energy expenditure is through a change in PAEE, although the change in TDEE is based on the magnitude of PAEE and the degree of behavioral and metabolic compensations that occur. Below we discuss possible ways that exercise can affect TDEE, not solely based on the net cost of exercise itself, but also due to the effects on non-PAEE. 

### 5.1. Physical Activity/Exercise Effects on TDEE

The energetic cost of exercise is a function of the mode, intensity, and duration of the activity. Considerable research has explored the impact of both acute and chronic PAEE on TDEE. At issue here, is whether or not physical activity or exercise can produce meaningful increases in TDEE. As discussed earlier, one cannot assume that an increase in PAEE will yield a predictable linear increase in TDEE. Pontzer [21,23] suggests that, based on the Constrained Energy Expenditure Model, the high prevalence of obesity is attributable to high food availability and intake and a limited capacity for elevating energy expenditure. However, there are challenges to this model such as laboratory-based studies that show habitual vigorous exercise in both young [34] and older adults [2] is associated with higher rather than lower REE. Although it is apparent that increased ExEE may be less effective in producing weight loss compared to calorie-restricted diets, we propose that following weight loss, increased PAEE via exercise can adequately increase energy flux to minimize weight regain. We further suggest that for most individuals who have lost weight by dieting and are seeking to maintain the weight loss, it is more likely that the true constraint on TDEE of PA is that the level of activity is insufficient to adequately increase TDEE rather than so high that it constrains TDEE. Indeed, changing from a sedentary lifestyle to an active lifestyle and regular exercise could significantly raise the metabolic scope (TDEE/REE), for example from 1.4 to 1.75 for many individuals, because the increase in physical activity from a sedentary state remains below the constraining threshold for TDEE. There may be a specific level of PAEE required for each individual necessary to generate sufficiently high energy flux to regulate energy balance at a healthy body weight. While data are severely limited, a number of studies [5,10,32,35] suggest that achieving a metabolic scope of approximately 1.7–1.8 may be an appropriate first target for individuals in the weight-reduced state. Clearly, more work is required to identify the metabolic scope that could become an appropriate population recommendation for weight loss maintenance and/or seek to understand how to best individualize metabolic scope values based on personal characteristics of the individual. 

### 5.2. Acute Endurance Type Exercise and Energy Flux

Understandably, energy expenditure increases during a bout of endurance exercise, which contributes to energy flux on that day. However, there are misconceptions as to the actual energetic cost of the activity. When calculating TDEE by summing REE, TEF, and PAEE, one must realize that the net energy cost of the activity (PAEE minus REE) should be used. The gross expenditure includes the REE during the duration of the exercise bout, or stated another way, the gross cost includes the energy expenditure during that time period, even if the individual had rested instead of exercised. To use the gross cost is to overestimate the contribution of an acute bout of exercise to the total energy flux. This overestimation is greater with longer, less intense physical activity, which includes a greater proportion of the gross energy expenditure as REE. 

There are also misconceptions as to the ability of an acute exercise bout to cause a prolonged elevation of post-exercise energy expenditure, the so-called excess post-exercise oxygen consumption (EPOC), thus adding to TDEE (and energy flux) beyond the net cost of the exercise bout itself. Numerous studies have been conducted to examine the effect of varying modes, intensities, and durations of exercise on EPOC. The results are not entirely concordant, but there is general agreement that for mild and moderate intensity endurance exercise (30–60% of maximal oxygen uptake (VO_2max_)) lasting 30–60 min the total post-exercise energy expenditure above pre-exercise resting values is small—likely in the range of 42–84 kJ (10–20 kcal) [36,37]. Importantly, exercise bouts of this nature are characteristically performed by the general public and many who initiate an exercise program during or after weight loss. In a series of well-designed experimental studies, Bahr et al. [38] demonstrated that exercise intensity has a much greater impact on EPOC than does exercise duration. This makes sense given high intensity exercise is a significant metabolic perturbation requiring a longer period for homeostatic recovery. Still, the impact on TDEE is small relative to the exercise itself. For example, Phelain et al. [36] showed that a high intensity exercise bout (75% VO_2max_) performed for approximately 50 min with a net energy cost of 2090 kJ (500 kcal) resulted in only additional 167 kJ (40 kcal) expended above resting values during the 3 h following cessation of exercise. Hunter et al. [39] found a single bout of high-intensity interval exercise increased non-ExEE by an average of 418 kJ (100 kcal) during the 22 h following exercise, compared to an additional 250 kJ (60 kcal) following 60 min of moderate-intensity (50% VO_2peak_) continuous aerobic exercise. While the magnitude of EPOC has not been well studied in the weight reduced state, it seems likely that the contribution of EPOC to daily energy flux is small, especially for low and moderate intensity exercise typically performed by individuals previously naïve to exercise. Note however, Bahr et al. [38,40] reported that a significant portion of the elevated post-exercise energy expenditure resulted from fatty acid-triacylglycerol cycling that could be quite beneficial in regard to improving metabolic flexibility and non-exercise fatty acid oxidation. 

### 5.3. Impact of Acute High Intensity Interval Training (HIIT) on Energy Flux 

There is considerable interest in high intensity, intermittent exercise training (HIIT), particularly as a low-volume, time-efficient alternative to endurance exercise. While the positive physiological adaptations associated with HIIT are well established [41,42,43], the influence of HIIT on energy flux and maintenance of lost weight is less clear. As is the case for acute exercise, misconceptions surround the ability of HIIT to sustain elevations in energy expenditure. To address these misconceptions, Sevits et al. [44] investigated the effect of a single session of HIIT (five 30 s sprints on a stationary cycle ergometer with a 4-min rest period between each sprint) on TDEE using a whole-room calorimeter. The net cost of the 22 min exercise bout (sprints plus rest intervals) averaged ~940 kJ (225 kcal), which was attributed entirely to the ExEE and the EPOC immediately following exercise completion. REE measured the following morning was unaffected by HIIT. Richards et al. [45] also showed that six sessions of HIIT spread over 2-weeks had no effect on REE. Thus, while EPOC is greater in HIIT vs. continuous exercise, elevations in post-exercise energy expenditure are not sustained through a 24 h period and must be repeated to maintain high energy flux. 

### 5.4. Impact of Chronic Endurance-Type Exercise on Energy Flux

There are numerous central and peripheral adaptations to endurance exercise training such as skeletal muscle mitochondrial biogenesis, increased oxidative enzyme abundance and activity, changes in futile cycling, changes in sympathetic nervous system activity, alterations in substrate utilization, etc. that improve metabolic flexibility in response to various dietary and exercise perturbations. Possibly these exercise-induced adaptions could affect energy flux independent from the energy expenditure of each exercise bout. Numerous cross-sectional studies [2,4,34,46] but not all [47] have reported higher REE values in endurance-trained compared to less well-trained individuals. VO_2max_ has been found to be positively associated with REE during high flux, but not low flux conditions [4]. Given the observational design of these studies, it is impossible to adequately control for possible confounders and causality cannot be inferred. The association of REE with VO_2max_ could result from the carryover effects of acute strenuous exercise bouts performed a day or two prior to the REE measurement, rather than due to exercise training adaptations per se. Bullough et al. [4] have previously shown the higher REE in highly-trained endurance cyclists compared to untrained individuals was largely the effect of several days of acute strenuous exercise accompanied by high energy intake, i.e., high flux, necessary to maintain energy balance. REE was significantly reduced when these athletes underwent a sedentary condition for several days and energy intake was simultaneously reduced to maintain energy balance (low flux). Thus, it is difficult to separate the effects on REE of training per se versus the last bout(s) of exercise interacting with TDEI. 

An important regulator of REE is the sympathetic nervous system [48]. Sympathetic support of REE is quantified as the magnitude of decrease in REE during beta-adrenergic receptor blockade [48,49] or during inhibition of sympathetic activity [50]. A greater decline in REE during this pharmacological manipulation is indicative of greater sympathetic support. In a state of high energy flux, sympathetic support of REE is appreciable but becomes negligible when energy flux is decreased via abstention from habitual exercise combined with decreased energy intake [2]. While the relationship between high energy flux and sympathetic support of REE has been well described, the physiological mechanisms responsible for this relationship are complex.

Sympathetic support of REE is determined by a combination of two factors: sympathetic activity and the thermogenic response to beta-adrenergic stimulation [51]. In a state of high energy flux, tonic sympathetic activity is high, as indicated by circulating catecholamine concentrations [4] and skeletal muscle sympathetic nerve activity (MSNA) [2]. However, sympathetic activity is also positively associated with whole body fat mass and abdominal fat [50,52]; leptin has been hypothesized to be the peripheral endocrine signal mediating the association [51,53]. Indeed, when transitioning from chronic high flux to a brief period (< 1 week) of low energy flux, both circulating leptin concentration and MSNA are decreased in the absence of changes in body and/or fat mass [2]. In endurance athletes, presumably in a chronic state of high energy flux, when matched with sedentary adults for total and abdominal fat mass, MSNA is higher [50]. Thus, it appears that the influence of energy flux on sympathetic activity may be independent of fat mass. 

While sympathetic activity is high in habitual exercisers and also in sedentary adults with overweight/obesity, the sympathetic support of REE is much lower in sedentary adults [49]. This can be partially attributed to the second determinant of sympathetic support of REE—the thermogenic response to beta-adrenergic receptor stimulation. Compared with endurance trained athletes, the increase in energy expenditure above REE during intravenous administration of the beta-adrenergic receptor agonist, isoproterenol, is lower in sedentary adults with overweight and obesity [54,55]. This decreased thermogenic responsiveness is mediated in part by oxidative stress [54] and decreased beta-adrenergic receptor sensitivity [55]. Thus, in a state of high energy flux mediated by vigorous habitual exercise, sympathetic support of REE is appreciable because of elevated sympathetic tone combined with upregulated beta-adrenergic receptor responsiveness. In contrast, in high energy flux mediated by obesity, sympathetic support of REE is low despite elevated sympathetic tone; this low support can be attributed to decreased beta-adrenergic receptor responsiveness. The increased sympathetic tone without the improved beta-adrenergic receptor responsiveness characteristic of obesity is associated with metabolic dysfunction and increased risk for cardiovascular disease [56].

Beyond REE, the sympathetic nervous system also contributes to other components of TDEE, including TEF (see Section 5.6) and PAEE. With respect to the latter, while there are some reported observations of lower VO_2_ during standardized exercise under conditions of beta-adrenergic receptor blockade [57], these observations are not consistent [58,59,60] and the influence of habitual activity and/or energy flux has not been described. 

In addition to the hormones (and neurotransmitters) associated with sympathetic activation, there are several other thermogenic hormones that may also contribute to TDEE, and therefore also the regulation of energy flux. For example, the synergistic action of catecholamines and the thermogenic thyroid hormone, triiodothyronine (T3) has been well described [61]. However, in the context of energy flux, we believe the contribution of T3 to energy expenditure to be minor. Circulating T3 is a very sensitive indicator of changes in energy balance [62,63]. Given that energy balance is a defining characteristic of energy flux, then the relevance of T3 to energy flux is primarily as a tool with which to confirm energy balance. Indeed, there are several examples within the literature in which T3 has been used in this capacity [2]. Similarly, leptin and insulin have both been linked with sympathetic activation [51] and have both been shown to exhibit thermogenic properties. As described previously, when transitioning from chronic high flux to a brief period (< 1 week) of low energy flux, circulating leptin concentration is decreased [2]. 

The exercise-training related elevations of REE in the above studies pose a challenge to the constrained TDEE model [23]. The apparent discrepancy may arise from exercise training characteristics of the participants in the studies discussed above, versus lower intensity occupational activity characteristic of many participants in ecological studies. Alternatively, the above studies were laboratory-based investigations, as opposed to observational reports of free-living humans. 

### 5.5. Impact of Resistance Exercise on Energy Flux 

It is generally recognized, that the net energy cost of resistance exercise bouts is typically lower than an equivalent amount of time spent in moderate intensity endurance exercise. For most weight lifting bouts, more time is spent recovering between sets than the time spent performing the number of repetitions within a given set. Thus, this mode of exercise might appear to be of lower importance for individuals seeking to increase daily energy flux following weight loss. However, there are benefits of resistance exercise that may be overlooked. Strenuous resistance exercise may result in a prolonged elevation of metabolic rate following exercise cessation, possibly up to 15 h later [64,65], which could add to the net cost of the actual exercise bout. Note, however, the magnitude of this effect on energy flux is likely to be lower for individuals who more commonly engage in less strenuous exercise, i.e., lower volume exercise due to less total weight lifted, fewer repetitions, and longer rest intervals between sets. 

Resistance exercise training is associated with increases in fat-free mass (FFM), which, if sufficiently large, may contribute to increases in REE. Note however, that the internal organs rather than skeletal muscle are the major contributors to REE. Resting skeletal muscle is estimated to expend only ~55 kJ/kg (~13 kcal) over a 24 h period [66], thus a substantial increase in muscle mass would appear necessary to produce a meaningful increase in REE. It seems doubtful that resistance exercise regularly performed by most individuals in the weight-reduced state would be sufficient to increase REE. Nevertheless, the impact of fat-free mass may be important beyond any impact on REE. FFM has been identified as an important regulator of energy intake, which could have significant implications for weight maintenance in the weight reduced state [67,68,69]. This notion is discussed further in Section 6.1.

### 5.6. Impact of Exercise on the Thermic Effect of Feeding 

Although the TEF accounts for a relatively minor proportion of TDEE (~10%) [70,71], over an extended period, small changes in TEF can have biologically relevant effects on energy balance. In this regard, the influence of habitual exercise on TEF has received much attention. Definitive conclusions remain elusive due to tremendous inconsistencies in experimental methodology, such as variation in meal size, macro-nutrient composition, and training/activity status of study populations. Still, compared with sedentary adults, many studies have shown TEF to be appreciably greater in habitual exercisers [72,73,74,75,76,77]. Importantly, this favorable consequence of habitual exercise appears evident irrespective of whether the exercise modality comprises endurance [73,74,75] or resistance training [76]. In addition to cross-sectional data, a select few intervention studies have also demonstrated increased TEF following short-term exercise training in previously sedentary adults [78,79]. Proposed physiological mechanisms for the greater TEF in habitual exercisers include increased fat free mass [70], augmented support from the sympathetic nervous system [74,75], and greater insulin sensitivity [77]. 

Pertinent to the current review, in adults who have lost weight through dietary restriction, absolute TEF is decreased due to lower energy intake [80,81]. This reduced contribution to TDEE may encourage positive energy balance and eventually failure to maintain weight loss. Consistent with our prevailing argument, increased energy flux may be one strategy by which high TEF could be maintained. High rates of energy expenditure, achieved through regular exercise, may contribute to greater TEF. Further, the magnitude of TEF is, in part, determined by the caloric content of the meal: higher energy intake leads to higher TEF [82,83]. Thus, in a state of high energy flux, daily dietary intake is also high, and therefore high TEF may be maintained. While this idea is intuitively appealing, preliminary support is not forthcoming as one pilot study has shown TEF to be largely unaffected by manipulations of energy flux following weight loss [10]. Theoretically, because postprandial thermogenesis is higher for protein than either carbohydrate or fat [84,85], higher protein consumption could contribute to higher energy flux and better weight loss maintenance. Several studies suggest that higher protein intakes following weight loss are associated with less weight regain [86,87], but this may be the result of the higher satiety value of protein rather than its higher thermic effect. The possible contribution of higher protein intake to energy flux should be studied further. 

## 6. Is Energy Flux Important for Weight Loss Maintenance and if so, How?

### 6.1. Physiology of the Weight Reduced State

Active weight loss is characterized by an energy deficit, while weight loss maintenance is characterized by a return to energy balance at the reduced body size. These are different physiological states, thus warranting a brief discussion of each. Those interested in a more thorough examination of the metabolic changes that result from the different phases of weight loss and the metabolic differences between active weight loss and the weight loss maintenance state are referred to an excellent recent review of these topics by Muller et al. [88].

Dietary restriction leading to weight loss is characterized by reduced energy expenditure, mostly due to loss of respiring tissue mass, but also due to adaptive thermogenesis (AT), which historically has referred to the changes in REE and nonREE independent of changes in fat-free mass (FFM) and fat mass (FM). Adaptive thermogenesis in response to active weight loss is present when REE and/or nonREE are reduced to an extent greater than can be explained by loss of fat mass and fat-free mass. Muller et al. [89] have shown that considerable individual variability exists in the magnitude of AT in response to calorie restriction and importantly, that when the composition of the FFM changes are accounted for, i.e., changes in the mass of skeletal muscle, liver, kidney, adipose tissue, etc., the magnitude of AT is quite small. In their study [89], in response to 3 weeks of severe energy restriction (50% of initial energy requirements that produced a mean weight loss of 6 kg), the mean reduction in REE was 266 kcal/d. However, only 60 percent of study participants exhibited AT and the mean AT was determined to be only 72 kcal/d. This suggests that much of the decline in energy expenditure that accompanies energy restricted-weight loss is predictable based on changes in the composition of the FFM and also that considerable individual response variability exists. Regardless of the magnitude of AT, it is evident that active weight loss usually results in a decrease in TDEE due to reductions in 1.) REE, 2.) TEF, as less food energy is consumed, and 3.) PAEE [70]. The latter declines as less energy is required to move the lower body mass, and as skeletal muscle work efficiency increases. [71,72,89]. 

Maintenance of lost weight is characterized by a return to energy balance without further weight loss or gain. Still, in the weight loss maintenance state, TDEE remains lower than prior to weight loss. REE remains reduced, but this phenomenon is largely explained by the composition of the FFM, i.e., there is less contribution of AT to the lower REE [88]. Also, PAEE declines, presumably resulting from less movement and the fact that less energy is required to move the lower body mass. Importantly, the increased skeletal muscle work efficiency continues during weight loss maintenance such that less energy is expended for a given low intensity physical activity [71,72]. Rosenbaum et al. [72] have estimated that as much as one-third of the decline in PAEE with diet-induced weight loss occurs as a result of this increased skeletal muscle work efficiency. Hill and colleagues have proposed the usefulness of the energy gap concept for weight loss maintenance [90]. Using whole-room calorimeters, they quantified the reduction in total energy expenditure that occurs following weight loss and termed this value the energy gap, which was estimated to be ~84 kJ/kg (~20 kcal/kg) of body weight lost (~840 kJ/day or 200 kcal/d for a 100-kg person losing 10% of body weight). 

Large inter-individual variability exists in the contributions of AT vs behavioral adaptations (e.g., decreases in PAEE) to weight loss-induced declines in TDEE. Why does one individual exhibit a decline in REE with weight loss that persists into the weight-reduced state that continues to exceed the loss of respiring mass, while another shows no such AT? Does this difference in AT suggest that the targets of energy flux best suited for limiting weight regain should be different in these two individuals? Would different energy flux targets and/or exercise approaches be warranted based on the magnitude of behavioral adaptations (decline in PAEE due to decreased NEAT and/or ExEE) versus contributions to the energy gap of AT? These questions appear largely unanswered at this time, but it would seem that regardless of the etiology of the lower TDEE following weight loss, regular exercise to increase energy flux could decrease the probability of weight gain by some or all of the following depending on the individual: increasing PAEE [10], increasing REE and offsetting AT to some extent [10], enhancing appetite regulation [28], increasing or preserving FFM and minimizing collateral fattening [91], and offsetting skeletal muscle AT as recently reported by Rosenbaum et al. [92]. They found that resistance exercise training significantly attenuated the increase in skeletal muscle work efficiency that accompanied weight loss. Possibly, knowing the magnitude of the increased skeletal muscle work efficiency that occurs with weight loss, can help individualize the types of physical activity (mode, intensity, and duration) that can best reverse this contribution to the decline in TDEE. Given that some individuals who initiate an exercise program compensate by decreasing NEAT (behavioral adaptation), it may be important to increase energy flux by both intentional increases in ExEE and NEAT, an approach used to enhance energy flux in an experimental study by Paris et al. [10]. Clearly, more research is required to better understand the unique behavioral and AT responses to weight loss that could then help tailor approaches to increasing energy flux best suited to personalized weight loss maintenance. 

To further add to the challenge, the energy gap is associated with several physiologic changes that provide strong biologic incentive to re-establish the body’s equilibrium at the original higher levels of body weight, body fat, and energy flux. This leads to a mismatch between energy expenditure and hunger, such that energy desired (influx) is greater than energy required (efflux). The increase in hunger is thought to result from reduced circulating leptin, insulin, anorexigenic gut peptides including amylin, cholecystokinin, peptide YY, etc., and increases in the orexigenic gut peptide, ghrelin [93]. Diet-induced energy deficits result in decreased adiposity, but FFM is also lost. Dulloo et al. [68], Blundell et al. [67], and Stubbs [69] posit that the increased hunger and physiologic drive to regain lost weight that persist in the weight loss maintenance state are more likely the result of a decrement in FFM than fat mass. Similar to how reduced body fat stimulates appetite, they suggest the loss of FFM also increases appetite so as to defend against losses of functional organ tissue and skeletal muscle needed to ensure human survival. During weight regain, the rate of fat mass re-accumulation typically exceeds that of FFM, thus hunger and energy intake increase until the FFM has reached its approximate pre-diet level. Possibly, this so-called ‘collateral fattening’ may even lead to overshoot with increases in fat mass that exceed the initial pre-diet adiposity level until the FFM is restored [91]. The feedback mechanisms and signaling molecules that connect FFM alterations with appetitive changes have not been elucidated, nor has the contributions to the putative feedback system of different components of FFM including skeletal muscle and internal organs. Nevertheless, the notion that the body maintains a FFM ‘memory’, suggests that minimizing the FFM loss while dieting and optimizing the rate of FFM regain during the weight-reduced state may be critically important in minimizing fat regain. Resistance exercise and even weight bearing endurance exercise that contributes to high energy flux may be especially important in this regard. 

The elevated hunger associated with peripheral signals that persist in the weight loss maintenance state [94] suggest the possibility that due to evolutional biology, there are limits on the ability of many humans to permanently reduce energy intake. Could the common experience of periodic food inadequacy and energy conservation in early humans that resulted in increased biologic drive for food carry over to modern times, such that a low flux state following weight loss results in the same drive for food experienced by our early ancestors? If so, maintaining permanent weight loss requires addressing the energy gap through 1) maintaining a low energy intake to match the reduced energy expenditure (low flux); 2) increasing energy expenditure by increasing physical activity to make up for the loss of energy expenditure due to weight loss (high flux); 3) a combination of achieving permanent changes in both energy intake and physical activity. 

Many obesity treatment programs focus largely on the first strategy of maintaining permanent reduction in energy intake, and most are unsuccessful in producing long-term weight loss maintenance. This approach (achieve energy balance at low energy flux) requires extraordinary willpower and vigilance to fight metabolic, behavioral and environmental pressures to regain. Since these pressures do not seem to dissipate over time [95], few people can maintain such willpower over the long term given the highly palatable and accessible food supply. Filling the energy gap with physical activity would seem to be the optimum strategy. However, efforts to produce significant sustained increases in physical activity have been difficult to achieve. It has been estimated that maintenance of significant weight loss of 10% or more may require ~40–80 min/d of moderate or vigorous intensity physical activity [96,97,98]. The third strategy, that weight loss maintenance could be attained by a combination of increasing physical activity and modification of aspects of food intake (e.g., energy density or portion size) that result in eating less without conscious effort. This approach holds promise and is discussed in a recent review [9] but has not been vigorously tested.

### 6.2. The Role of High Flux in Maintenance of Lost Weight

**Figure 4:**
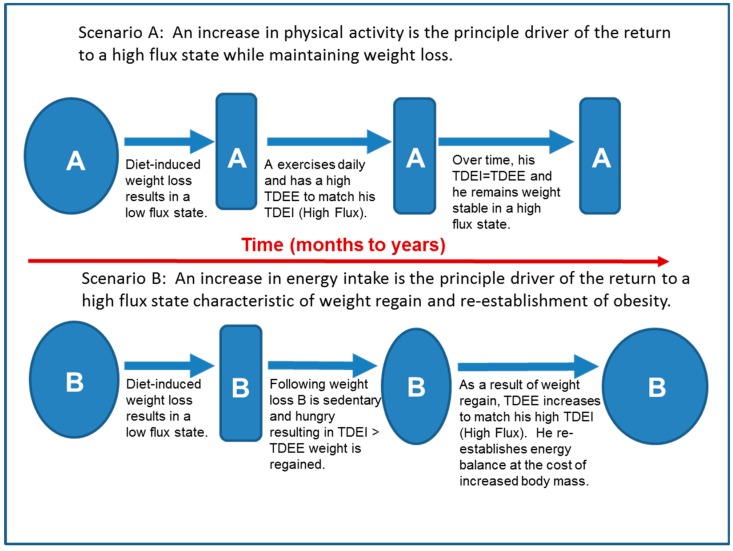
Two scenarios describing the different ways that a high energy flux state could be achieved following weight loss, with scenario A resulting in maintenance of lost weight, while scenario B results in a return to the obese state.

In individuals predisposed to excessive weight gain, the high flux state characteristic of obesity appears to be driven primarily by high food availability and the attendant high TDEI. One might then conclude that following weight loss, the best approach to maintaining weight loss is to sustain a low TDEI. However, we have argued that a low energy flux state is not sustainable by most individuals because, as shown in Figure 4 below, the increased hunger in an environment of high food availability results in an increase in TDEI that serves as the principle driver of the return to a high flux state at a higher body mass (Scenario B). Instead, we propose that following weight loss, increased PA should be the principle driver of the return to a sustainable high flux state, which could theoretically be achieved without a significant increase in adiposity (Figure 4, Scenario A).

There is some evidence that increased physical activity adequate to elevate energy flux following weight loss is a key determinant of long-term weight loss maintenance in both human [96,99,100] and animal models [101]. Observational studies of participants enrolled in weight loss registries in the U.S.A. [102] and Greece [103] have shown significantly higher levels of physical activity (ostensibly higher energy flux) in weight loss maintainers compared to weight re-gainers. In a recent case-control study, Ostendorf et al. [35] found significantly higher levels of PAEE in those individuals who maintained weight loss (WLM) compared to controls with overweight or obesity (OC). However, in line with the thesis of this paper, TDEE was not different between the two groups, owing to higher non-PAEE in the OC. Thus, both groups exhibited similar levels of energy flux but with differences in body mass and metabolic scope (TDEE/REE for WLM = 1.75; OC = 1.55). 

Few experimental studies have actually identified the impact of high flux on factors related to weight loss maintenance. Paris et al. [10] conducted a randomized, cross-over feasibility experiment in which individuals with obesity underwent modest energy restriction to produce a 7% weight loss over several months followed by 3 weeks of weight stabilization at their reduced body weights. Following stabilization, they were assigned in random order to complete 4-day high flux and 4-day low flux conditions with a weight stable washout period in-between. For high flux, the individuals completed a monitored exercise bout each day (net ExEE of 2092 kJ/day (500 kcal/d) at 65% VO_2max_) with additional walking to achieve a metabolic scope of 1.7 (TDEE = REE × 1.7). Participants were provided with an equivalent amount of food to maintain energy balance during each 4-day condition. Low flux was a sedentary condition in which participants did not exercise and engaged in limited physical activity. Again, all food was provided with energy intake set at RMR × 1.35 to match the low TDEE in order to maintain energy balance. The exercise and additional walking attenuated the energy gap, but in addition, average daily REE was significantly higher (8060 kJ/day; 1926 kcal/d) during the four days of high flux compared to the four days of low flux (7730 kJ/day; 1847 kcal/d). Importantly, during high flux the study participants reported significantly lower hunger and greater fullness compared to their days spent in low flux, despite being in energy balance across the two different flux conditions. These data support the concept that at higher levels of energy flux owing to greater physical activity, appetite is likely to be regulated more accurately to match TDEE and minimize weight gain, even in those individuals who are at a reduced body weight. A recent experimental study by Hagele et al. [5] lends support to the notion that high energy flux results in better appetite regulation compared to a low flux state, although study participants (*n* = 16) were mostly of normal weight (only one individual exhibited a BMI > 30 kg/m^2^ and participants were not subjected to a weight loss program). Their better acute appetite regulation while in high flux compared to low flux was associated with lower circulating concentrations of the orexigenic hormone, ghrelin and higher concentrations of the anorexigenic hormone, GLP-1. An obvious limitation of these studies is the short amount of time participants spend in the high and low flux states, which points to the need for experimental studies of a longer duration. 

## 7. What are the Important Research Questions That Should be Addressed?

A principal purpose of this paper is to bring greater awareness to the potential impacts of energy flux on the prevention and treatment of obesity and metabolic functioning. Consistent with that purpose, much of the concepts herein have not been rigorously tested, but rather seek to generate novel research directions to prove or disprove the concepts we have presented. Given the complexities of body weight regulation, we have undoubtedly oversimplified these concepts. Thus, we believe that future research in this field will need to span the translational research spectrum including basic/pre-clinical models, highly-controlled human feeding and exercise efficacy trials, pragmatic intervention studies to test effectiveness in “real-world” settings, and observational (especially prospective) studies conducted in free-living people. We have suggested that the concept of energy flux may be useful in understanding how and why obesity develops and why our long-term success in treating it is poor. 

There is still a need for consensus on definitions and measurement of energy flux. Various definitions and methodological techniques have been used, which may be limiting the pace of more innovative applications of the energy flux concept to human health and well-being. In particular, future research in this area should seek to standardize the conditions under which energy flux is measured (e.g., energy balance), the timeframe under which energy flux is determined and measured (e.g., hours vs. days vs. weeks), and how energy flux is similar to and different from energy expenditure. While we have proposed that relative energy flux should be quantified as TDEE/REE, possibly regression-based models that use REE, FFM, or other measures of respiring mass would be more valid/useful than the ratio method. It will also be important to determine the characteristics of physical activity (i.e., mode, intensity, and duration) that best increase TDEE and more accurately regulate appetite. Also, the potential effects of dietary macronutrient composition on energy flux is an area requiring further study. 

Numerous other questions remain to be answered with regard to the impact of energy flux on body weight regulation. Principal among them is a rigorous testing of the Constrained Total Energy Expenditure Model proposed by Pontzer et al. [23], and the environmental drivers (high-food availability and low need for physical activity) that may predispose modern developed populations to an obesity-induced high energy flux state. Future research in this area will help to define the complex and interacting relationships of physical activity, eating behavior, and body weight regulation. These concepts have major implications for the relative importance of personal behavioral change strategies (e.g., reducing energy intake and increasing physical activity) and environmental modifications for stemming the persistent obesity epidemic. Identifying the minimum, maximum, and optimum levels of energy flux at the individual level will be critical for developing personalized strategies to achieve long-term energy balance and weight stability. Finally, identifying factors that explain the substantial individual response variability in appetite, REE, non-PAEE, energy efficiency, etc. seen with changes in PA is of critical importance. 

## 8. Summary and Conclusions

Within the current environment, most people who voluntarily lose weight will need to subsequently increase their energy flux in order to reestablish energy balance and achieve a stable body weight. The two ways this can occur are increasing body weight (regaining) or increasing physical activity. The most popular strategy for obesity treatment is food restriction and there is great debate about the optimum diet for weight loss. Many researchers argue fiercely about the merits of different types of energy restricted diets in producing weight loss, while in reality, none are particularly effective at maintaining weight loss, suggesting that increased energy flux occurs from weight regain in these individuals. An alternative approach is increasing physical activity, filling the energy gap, and increasing energy flux without increasing body weight. Achieving the amount of exercise required to maintain lost weight in a high flux condition is a major challenge, but may be more feasible than sustained food restriction. There seem to be powerful biological processes and environmental factors opposing food restriction, but little evidence of biological opposition to a high flux state characterized by a metabolic scope of 1.7–1.8 achieved by way of increased physical activity. It may be possible to identify a threshold of energy flux that would need to be maintained to reduce the probability of excessive weight regain. We suggest that for most individuals, food restriction alone is not an effective long-term strategy for weight loss maintenance and that future research efforts should be focused on the interaction of diet and exercise in achieving high energy flux at a healthy body weight.
